# Colonization potential of endophytes from halophytic plants growing in the “Runn of Kutch” salt marshes and their contribution to mitigating salt stress in tomato cultivation

**DOI:** 10.3389/fmicb.2023.1226149

**Published:** 2023-08-29

**Authors:** Pramod K. Sahu, Zaryab Shafi, Shailendra Singh, Khushboo Ojha, K. Jayalakshmi, Jyotsana Tilgam, Nazia Manzar, Pawan K. Sharma, Alok K. Srivastava

**Affiliations:** ^1^ICAR-National Bureau of Agriculturally Important Microorganisms, Mau, Uttar Pradesh, India; ^2^Department of Biotechnology, Invertis University, Bareilly, Uttar Pradesh, India; ^3^ICAR-Directorate of Onion and Garlic Research, Pune, Maharashtra, India

**Keywords:** endophytes, halophytes, oxidative stress, ROS scavengers, salinity

## Abstract

Increasing soil salinity depreciates the quantity of the crop produce. Looking at the tremendous potential of plant-associated microorganisms in salinity stress mitigation, it would be very useful in exploring and deciphering salt-tolerant microorganisms from halophytic plants and their utilization in cultivated plants. With this aim, in the present study, four halophytic plants were taken from Rann of Kutch, and bacterial endophytes were isolated from different plant organs. These endophytes were characterized by plant growth and health promotion features. The molecular identification was done based on 16 s rRNA sequence similarity. It was found that the endophytic bacteria isolated from 4 different halophytes found sharing phylogenetic relatedness. Four potential endophytes *Alkalihalobacillus gibsonii* 2H2, *Achromobacter insuavis* 2H18, *Terribacillus halophilus* 2H20, and *Bacillus siamensis* 4H1 were tested in tomato for salinity stress alleviation. Changes in the levels of antioxidants were analyzed. Total chlorophyll, total phenolics, malondialdehyde, and proline content indicated reduced damage in the plant system due to salinity by the application of endophytes. All the treatments exhibited low levels of electrolyte leakage. The accumulation of enzymatic reactive oxygen species scavengers was assessed from the levels of peroxidase, catalase, superoxide dismutase, phenylalanine ammonia-lyase, ascorbate peroxidase, and guiacol peroxidase. The NBT and DAB staining confirmed the findings. The reduction in the accumulation of Na^+^ ions in tomato leaves was visualized using Sodium Green probes under CSLM and found to be lowest in *Terribacillus halophilus* 2H20 and *Bacillus siamensis* 4H1 inoculated plants. The endophyte *Terribacillus halophilus* 2H20 was the most promising isolate. The colonization in tomato roots was confirmed using a cell tracker system. Results showed that the endophytes were found to have salinity stress mitigation traits. The efficiency could be further improved with the combination of other endophytes tested earlier.

## Introduction

Global food security is now a significant concern due to the steadily expanding population. The 2020 Global Agricultural Productivity (GAP) Report estimates that to satisfy the needs of 10 billion people for food in 2050, the GAP must rise by 1.73 percent per year (GAP Report, 2020). Plant health promotion is a very important aspect of agriculture and there is a constant demand for quality products in the market. The consumers are also increasing in an inappropriate ratio, in comparison to the production. An increase in the problem soil is a challenge for the crops to produce high quantity and quality products with ever-shrinking arable lands (Sharma and Singh, 2017) The soil quality has significantly decreased as a result of climate change and human activity, which has reduced agricultural productivity. Soil salinization is one of the utmost detrimental elements that contribute to the deterioration of arable land among the different risks to soil functions (Ilangumaran and Smith, 2017; Otlewska et al., 2020). One of the biggest issues facing the world today that has a detrimental impact on crop output is salinity. Salinity hampers plant growth by several ill effects such as water stress, cytotoxicity from excessive amounts of sodium (Na^+^) and chloride (Cl^−^) ions, nutritional imbalance, etc. (Isayenkov and Maathuis, 2019). According to estimates, salinity now affects more than 20% of all agricultural land globally, and by 2050, that percentage will rise to 50% (Otlewska et al., 2020). Low water potential caused by excessive amounts of salt build-up in the soil hinders plant growth and development by generating ion toxicity, nutritional shortage, and inhibition of photosynthesis (Sahu et al., 2021).

It is well recognized that soil salinity inhibits plant growth first by osmotic stress and then by ion toxicity. Reactive oxygen species (ROS) produced by salt stress could cause oxidative damage and significantly impede plant growth and development (Singh and Jha, 2016). By limiting root growth, nutrient uptake, and metabolic activities, soil salinity reduces crop output (Chung et al., 2019). As a result of poor root growth and development, the active rhizosphere zone is diminished, affecting efficient nutrient uptake. Salinity stress also has an impact on physiological, morphological, and biochemical processes, resulting in a reduction in crop biomass and production (Yoon et al., 2009). By competing with K^+^ ions, more Na^+^ and Cl^−^ ions cause ionic imbalances and ion toxicity. Chlorosis and necrosis are caused by ion toxicity, which disrupts a variety of physiological processes in plants (Almeida et al., 2017). By competing with K^+^ for binding sites and interfering with K^+^ homeostasis, Na^+^ suppresses crucial cellular processes and enzymatic activities that depend on K^+^ for proper operation. To protect the plant from oxidative damage, however, plants produce antioxidant enzymes, which quench excessive amounts of ROS. These enzymes include superoxide dismutase (SOD), peroxidase (POD), polyphenyl oxidases (PPOs), and catalase (CAT). Also, the plant growth promoting endophytes (PGPEs) can make antioxidant enzymes like PODs, SODs, CATs, and PPOs, which get rid of ROS that plants make too much of when they are stressed by salinity (Afridi et al., 2019).

There have been significant attempts undertaken to increase food yield in salty soils via different approaches (Derbyshire et al., 2022), however, the microbe-mediated approach of enhancing productivity and quality would be a rather easy and sustainable option that could be extended to any crop and cultivars of commercial importance. In addition, there may be certain regulatory concerns associated with the creation and cultivation of genetically modified crops (Kumar et al., 2020). It has been proposed that maintaining low Na^+^ concentrations in the cytoplasm and cytosol and maintaining high cytosolic K^+^ concentrations are necessary for plant life under salinity stress. The plant-associated microbes have a huge potential for reducing excess Na^+^ ions from entering the active cell metabolism and mitigating the secondary effects of salinity (Sahu et al., 2021). Endophytic microorganisms can mitigate the negative impacts of salinity in plant systems through adaptations at various morphological, biochemical, and physiological processes (Gupta et al., 2023a).

Endophytic bacteria control the expression of genes, which leads to the production of different phytohormones, antioxidant enzymes, siderophores, volatile organic compounds, ROS-scavenging enzymes, and other chemicals (Sahu et al., 2021). A natural method for enhancing a plant’s growth and output under salt may be to use bacterial endophytes as its second genome (Ali et al., 2022). The current study looked carefully at the various mechanisms by which endophytic bacteria can control plant growth and alleviate the effects of salt stress.

The Runn of Kutch is a large salt marsh spanning from the State of Gujrat in India to Sindh Province in Pakistan, comprising two parts: the ‘Great Rann’ and the ‘Little Rann’. Being close to sea level, it is flooded with water, creating a wetland in the rainy season and a salt marsh when the water dries up (Britannica, 2023). Since the salt content is high, plants adapted to hypersaline environments can grow in these salt marshes. Halophytes are plants adapting to an extremely hypersaline environment, and their microbiome helps the host plant survive and perform growth and reproduction under such conditions (Gao et al., 2016). Exploring these endophytes for salinity alleviation in other crops growing in the salt-affected area could be useful in mitigating the ill effects of salinity. With the present leads available, focused further study in this area is required, notably on exploring potential stress-alleviating endophytes and their deployment in other food crops. Thus, in the present study, we have explored the plant growth-promoting endophytes from four halophytic plants growing in the Runn of Kutch and studied their mechanisms in tomato plants for providing protection from salt stress. Hence, the main purpose of this research was: (i) to evaluate the potentiality of endophyte isolates to perform Plant Growth Promoting (PGP) features *in vitro* in the presence of different salt levels, (ii) to study the efficiency of endophyte isolates to colonize internal plant tissues, (iii) to investigate the Endophytic treatment effect on stressed plant growth, and (iv) to evaluate the influence of the bacteria on membrane integrity, ROS management, ion equilibrium, chlorophyll contents, and malondialdehyde (MDA) and hydrogen peroxide (H_2_O_2_) accumulation.

## Materials and methods

### Isolation of endophytes

The bacterial endophytes were recovered from four distinct halophytic plants, viz. (1) *Suaeda palmirosa,* (2) *Suaeda nudiflora,* (3) *Aeluropus* sp., and (4) *Cressa cretica* from Runn of Kutch. The root and shoot portions were separated and cleaned under running water. Furthermore, the surface sterilization was carried out using the technique described in Sahu et al. (2020) with slight modifications. The exposure to 2% sodium hypochlorite (NaOCl) was maintained for 30 s followed by three sterile distilled water wash. The residual toxicity of NaOCl was neutralized by dipping tissue in 2% sodium thiosulfate for 10 min, followed by three sterile distilled water washes. Using a sterile pestle and mortar, surface sterilized plant tissue was macerated in 12.5 mM potassium phosphate buffer. The dilutions 10^−2^ and 10^−3^ were prepared and inoculated on a nutrient agar medium by spread plating (Buck and Cleverdon, 1960). In addition, anatomically different colonies were collected and purified for future use.

### Characterization for functional diversity

#### Assessment of salt tolerance of endophytes

The capacity of the chosen endophytes to tolerate NaCl was further assessed as per the protocol followed by Gupta et al. (2023b). The isolates were grown/cultured in NB media supplemented with various sodium chloride (NaCl) levels (0, 400, 800, 1,200, 1,600, and 2000 mM) for this, then incubated at 28 ± 2°C in a shaking incubator (at 150 rpm) for two to 3 days. Following incubation, metabolically active cells were examined using the spread plate technique (Buck and Cleverdon, 1960). The salt concentrations were tested up to 2000 mM, and as the classification of Larsen (1986), the strains that could easily survive salt concentrations of >800 mM in this study, were classified as moderately halophilic.

#### Exopolysaccharide production

The isolates were subjected to an exopolysaccharide production (EPS) bioassay. Selected strains were spot inoculated onto Nutrient agar plates (10 μL, 0.5 OD^600^) and incubated at 28 ± 2°C for 48 h. Mucoid development surrounding colonies was identified as an indication of the capacity of endophytic isolates to generate EPS. Positive isolates were chosen for further testing. In liquid culture, the strains capable of generating EPS were evaluated for quantitative EPS production. For this purpose, 100 mL of LB medium was inoculated with 100 μL of bacterial culture (0.5 OD^600^) and incubated at 28 ± 2°C at 100 rpm in a shaking incubator. After 96 h, the bacterial culture was centrifuged, and the supernatant was collected in a sterilized flask. Exopolysaccharide was precipitated in the supernatant by adding pre-chilled acetone (3:1 v/v). Freshly prepared exopolysaccharide was dried, and weight (mg) was measured (Latif et al., 2022).

#### Indole acetic acid

Luria Bertani (LB) broth with and without 5 g/mL tryptophan was used to study the synthesis of indole acetic acid (IAA). Cultures were centrifuged at room temperature for 10 min at 5000 RPM after 5 days of incubation at 28 ± 2°C. Two drops of orthophosphoric acid and 5 mL of Salkowski’s reagent were added to 10 mL of supernatant. The development of pink color suggested IAA production (Patten and Glick, 1996), and the degree of pinkness was measured by absorbance at 530 nm. The standard curve was used to correlate the amount of IAA produced by the isolates in μg mL^−1^.

#### Phosphate solubilisation

Using Pikovskaya’s agar medium, the endophytic isolates’ phosphorus solubilization was evaluated (Pikovskaya, 1948). Endophyte cultures were spotted on Pikovaskya agar plates (10 μL, 0.5 OD^600^) and incubated for 2 days at 28 ± 2°C. The appearance of a clean zone surrounding the colonies was indicative of successful phosphate solubilization.

#### Ammonia production test

The production of ammonia by endophyte isolates was assessed as previously described (Cappuccino and Sherman, 1992). The peptone water was supplemented with endophyte cultures, and the mixture was maintained at 28 ± 2°C for 72 h for growth. Once the sample had been incubated, 0.5 mL of Nessler’s reagent was added, and the presence of a yellow-brown tint was deemed positive.

#### Siderophore production test

The modified CAS agar technique was used to test siderophore production (Chrome Azurol S); (Schwyn and Neilands, 1987). After growing for 24 h, endophyte cultures were spotted onto CAS agar plates (10 μL, 0.5 OD^600^) and incubated for 3 days at 28 ± 2°C. The siderophore production was evident by the development of orange hallo around the colonies.

#### Hydrogen cyanide production

The production of hydrogen cyanide (HCN) was evaluated in accordance with Ahmad et al. (2008). Cultures were streaked on nutrient agar plates with 4.4 gL^−1^ glycine. The upper half of a Petri plate was covered with Whatman No. 1 filter paper that had been soaked in a solution of 2 percent sodium carbonate and 0.5 percent picric acid. The plates were covered and incubated for 6 days at 28 ± 2°C. The HCN production for 6 days was demonstrated by the color shift from yellow to orange-brown.

#### Molecular identification based on 16S rRNA

Molecular identification of the isolates was done as per the protocol described in Sahu et al. (2020). Briefly, 24 h old colonies of respective endophytes were taken for DNA isolation using Nucleopore gDNA Fungal/Bacterial Mini Kit (Genetix, New Delhi, India) following manufacturer’s instructions. The isolated DNA was used for conducting PCR for 35 cycles with 16S rRNA primers (PA 5′AGAGTTTGATCCTGGCTCAG-3′ and PH 5′-AGGAGGTGATCCAGCCGCA-3′). The PCR product was purified and sequenced by Sanger sequencing. The sequences were identified using EZbiocloud database^1^ and NCBI.^2^ Sequences were trimmed for quality and the contig was formed using the Bioedit program (Hall, 1999). Sequences of all the endophytes were aligned and compared using CLUSTAL-W with default parameters (Thompson et al., 1994). The neighbor-joining (NJ) method was used for constructing phylogeny (Saitou and Nei, 1987) using MEGA 10.0 (Kumar et al., 2018).

### *In vivo* assessment of salinity tolerance in tomato

#### Soil preparation and nursery

The four most promising isolates (2H2, 2H18, 2H20, and 4H1) were tested *in vivo* for their salt stress mitigating properties in tomato plants. For this *in vivo* trial, five kilograms of autoclaved soil were put into the plastic pots. Row and column randomization eliminated the variation within the pots. The nutrients were combined using urea, diammonium phosphate, and muriate of potash at a ratio of 100:50:50 kg NPK per acre. Three soaking and drying cycles were used to naturally compress the soil in these pots. The pots were watered to 80% of the field’s capacity, and 150 mM NaCl was used to maintain salinity. In a planting tray containing soil, sand, and cocopeat mixture (1:1:1), tomato seeds of the variety Pusa Ruby were planted and kept wet by misting them with water. Plants were transplanted after 30 days of sowing. The roots were gently rinsed and treated with the appropriate endophytic inoculant by root dipping. Each of the five treatments had four replications. The treatment details were as follows: 4H1, 2H2, 2H18, 2H20, and control. The roots were soaked in 0.5 percent carboxy methyl cellulose for 30 min before being transplanted into the pots with the inoculum (5 mL/L; 24 h-old 0.2 OD culture of respective endophytes). The experiment was repeated twice. Data was collected 45 days after transplantation (DAT), however, the plant’s length and dry weight were measured at 75 DAT.

#### Total chlorophyll content

The total chlorophyll concentrations were calculated by homogenizing 0.5 g of fresh leaves in 80 percent acetone. Using a spectrophotometer, the extract’s absorbance was determined at wavelengths 645, 663, and 470 nm. The concentration of total chlorophyll content was determined using the previously reported technique (Sadasivam and Manickam, 1996). The following formula was used to calculate the total chlorophyll content:

$$\mathrm{mg}\;\mathrm{total}\;\mathrm{chl}/g\;\mathrm{tissue}=20.2\;\left(A_{645}\right)+8.02\;\left(A_{663}\right)\times V/1000\times W$$

Where, A_λ_ = absorbance at specific wavelength λ (nm); V = final volume of chlorophyll extracted in 80% acetone, and W = fresh weight of tissue extract.

#### Total polyphenolic content (TPC)

For TPC analysis, methanolic extracts (1 mL) from tomato leaf samples were combined with water: methanol (1:1, 1 mL, v/v), distilled water (3 mL), and newly produced Folin-Ciocalteau reagent (0.5 mL) followed by vigorous mixing (Sadasivam and Manickam, 1996). 5 min of incubation was followed by the addition of 1 mL of 5 percent sodium carbonate. After 30 min, the absorbance was measured at 725 nm. TPC was determined as gallic acid equivalents (GAE) g^−1^ FW.

#### Extraction for enzyme assays

About 0.5 g of flash-frozen leaves in liquid nitrogen was homogenized in 12 mL of ice-cold phosphate buffer (100 mM; pH 7.0; 0.5 mM EDTA) containing 1.4 mmol L^−1^ -mercaptoethanol, using a mortar and pestle. The homogenate was centrifuged at 16,000 g for 15 min at room temperature to separate the supernatant, which was then utilized to conduct enzyme tests.

##### Phenylalanine ammonia-lyase (PAL) assay

The reaction mixture was prepared by adding 0.1 mol L^−1^ of phenylalanine (pH 8.7), 0.2 mol L^−1^ of phosphate buffer (pH 8.7), 0.2 mol of enzyme extract (0.2 mL), and distilled water (1.3 mL). The reaction was stopped by adding 0.5 mL of trichloroacetic acid (1 mol L^−1^) after 30 min of incubation at 32°C. At 290 nm, the absorbance was measured, and the activity was measured in mol of t-cinnamic acid (TCA) per g of formic acid (FW) (Brueske, 1980).

##### Superoxide dismutase (Sod)

The enzyme activity of SOD was measured by the ability of enzyme extract to stop the photochemical reduction of nitroblue tetrazolium (NBT) chloride. The reaction mixture contains 200 μL of enzyme extract combined with 100 mM phosphate buffer (pH 7.8), 3 mM EDTA, 1.5 m sodium carbonate, 2.25 m NBT, and 3 mM EDTA in a final volume of 3 mL. After adding 400 μL of riboflavin (2 mol l^−1^), the process was initiated by placing the tubes under two 15 W fluorescent lights for 15 min. After placing the tube in complete darkness to stop the process, the absorbance at 560 nm was measured. One unit of the enzyme extract lowered the absorbance by 50% in contrast to the control, which did not contain any enzyme extract (Fridovich, 1974).

##### Peroxidase (PO)

PO activity was measured in a reaction mixture containing 500 μL H_2_O_2_ (1 percent v/v), 1.5 mL pyrogallol (0.05 mol L^−1^), and 50 μL of an enzyme. At 420 nm, the change in absorbance was noted at regular intervals of 30 s for 3 min. U min^−1^ g^−1^ FW was used to express the enzyme activity (Hammerschmidt et al., 1982).

##### Ascorbate peroxidase (APX)

In this test, a mixture of ascorbic acid (0.25 mM), H_2_O_2_ (1.0 mM), EDTA (0.1 mM), and phosphate buffer (25 mM, pH 7.0) was used. Enzyme extract (0.2 mL) was added to this mixture to start the reaction, and after 60 s, a decrease in absorbance at 290 nm was observed. The enzyme activity was measured as U min^−1^ g^−1^ FW of leaf tissue (Nakano and Asada, 1981).

##### Catalase (CAT)

Catalase activity was assessed by measuring decomposition of H_2_O_2_ as per the method of (Aebi, 1984) at 240 nm. One unit (U) of catalase activity was defined as the amount of enzyme that caused an absorbance change of 0.001 per min under assay conditions. The reaction mixture contained 100 mM sodium phosphate buffer (pH 7.0), 30 mM H_2_O_2_, and 100 μL of crude extract in a total volume of 3.0 mL.

##### Guaiacol peroxidase (GPX)

GPX was measured as per the protocol given by Zheng and Van Huystee (1992) by recording the increase in absorbance at 470 nm. The reaction mixture consisting of sodium phosphate (10 mM; pH 6.0), H_2_O_2_ (0.3%, v/v), tetraguaiacol (1%, v/v), and enzyme extract (0.3 mL) was prepared. The enzyme activity was represented in terms of U min^−1^ mg^−1^ FW where one unit of enzyme catalyzes the oxidation of 1 μmol of guaiacol min^−1^.

#### Proline estimation

Proline content was measured as described in Sadashivam and Manickam (1996). The plant sample (0.5 g) was crushed in 10 mL 3% aqueous sulphosalicylic acid, followed by filtering with Whatman no. 2 filter paper. In 2 mL of filtrate, 2 mL glacial acetic acid and 2 mL acid ninhydrin were added and kept in a boiling water bath for a period of 1 h. The reaction was terminated by placing it in an ice bath. Further, toluene (4 mL) was mixed by stirring for 20–30s, and the solution was kept at room temperature for toluene layer separation. The upper layer was transferred to a cuvette, and the intensity of red color was measured at 520 nm using a UV–vis 1700 spectrophotometer, Shimadzu, Japan. Calculations were performed using a standard curve, and the values are shown as mg g^−1^ tissue.

#### Determination of lipid peroxidation

Malondialdehyde (MDA) measurements were used to calculate the quantity of lipid peroxidation (Heath and Packer, 1968). The root tissues from the arsenic-treated and control groups were divided into tiny pieces and crushed by adding 1 mL of 0.1 percent cold TCA. The samples were then put into new tubes and centrifuged at 10,000 g for 20 min at room temperature. A fresh tube was filled with 1 mL of supernatant and 1 mL of 20% TCA (containing 0.5% thiobarbituric acid and 0.01 mL 4% butylated hydroxyl toluene in ethanol). The tube was then incubated at 96°C for 35 min. The tubes were then put in an ice bath and left there for 5 min before being centrifuged at 10,000 g for 5 min. The supernatant’s absorbance was measured at 532 nm, and the absorbance at 600 nm was subtracted to adjust for non-specific turbidity. The amount of MDA was determined based on its molar extinction coefficient, which was 156 mmol L^−1^ cm^−1^, or mmol L^−1^ g^−1^ FW.

#### Electrolyte leakage

The total inorganic ions leaked out of the leaves were calculated as previously outlined by Khare et al. (2010). First, 0.5 g of leaves were taken in a boiling test tube containing 10 mL of deionized water and the electrical conductivity (EC) was measured (EC_a_). The contents were heated at 45°C and 55°C for 30 min each in a water bath and the EC was measured (EC_b_). Later, the controls were boiled at 100°C for 10 min and the EC was again recorded (EC_c_). The electrolyte leakage was calculated by using the formula:

$$\mathrm{Electrolyte leakage}\;\left(\%\right)=\left({\mathrm{EC}}_{b}-{\mathrm{EC}}_{a}/{\mathrm{EC}}_{c}\right)\times 100$$

#### Accumulation of reactive oxygen species

ROS accumulation in tomato leaves was assessed by the protocol discussed in Sahu et al. (2021). Briefly, H_2_O_2_ accumulation in tomato leaves was detected by 3,3-diaminobenzidine (DAB) staining (Sharma et al., 2008). Tissue was dipped overnight in DAB solution and brown color spots developed were imagined after chlorophyll removal by ethanol: glacial acetic acid (3:1) mixture. The superoxide radical accumulation was detected by overnight dipping in nitroblue tetrazolium (NBT) solution. Blue color spots developed were visualized after chlorophyll removal by ethanol:glacial acetic acid (3:1) solution.

#### Sodium accumulation in leaves

Accumulation of Na^+^ ions in tomato leaves was visualized using Sodium Green™, Tetra (Tetramethylammonium) Salt (Invitrogen, USA). In this study, the sodium fluorescence was captured using Confocal Scanning Laser Microscope as per the protocol given in Gupta et al. (2023a). Briefly, thin sections of tomato leaves of equal age and position were taken and stained with Sodium Green™ and incubated at room temperature in the dark for 30 min. The unbound dye was removed by washing with sterile distilled water and mounted on a glass slide for visualizing under a 488 nm channel in CSLM. The NIS element 3.2.3 program (Nikon, Japan) was employed for capturing images.

### Colonization of endophytes in tomato roots

The colonization was checked using CellTracker™ Blue CMAC Dye (7-amino-4-chloromethylcoumarin, Invitrogen, USA) following manufacturers’ instructions. The tomato seeds were surface sterilized as per the protocol used in Sahu et al. (2021). Seeds were soaked in sterile filter paper and respective cultures of four inoculants treated with CellTracker™ Blue CMAC Dye were inoculated in seeds after the emergence of radical (concentration of cells used 0.2 OD at 600 nm). In control seeds, all the steps were followed similarly except for adding inocula. After 48 h, the root surface was cleaned with sterile water thrice and cross sections were imaged under 405 nm laser in a confocal scanning laser microscope (Nikon A1 90i, Japan).

### Statistical analysis

Four separate experimental sets (*n* = 4) were used in all five treatments, which were carried out in a completely randomized block design (CRBD). The mean value of the replicates combined with the standard deviation was used to express the results for all experiments that underwent One Way ANOVA. The means were compared by Duncan’s Multiple Range test (DMRT).

## Results

### Isolation of endophytes

A sum of 52 endophytic bacteria were isolated from four halophytic plants. Nine isolates from *Suaeda palmirosa* (indicated by 1H), 15 from *Suaeda nudiflora* (indicated by 2H), 14 from *Aeluropus* sp. (indicated by 3H), and 14 were isolated from *Cressa cretica* (indicated by 4H). These colonies differed from each other in size, shape, color, surface texture, margin, amount of EPS production, and growth patterns.

### Functional diversity

**Figure 1:**
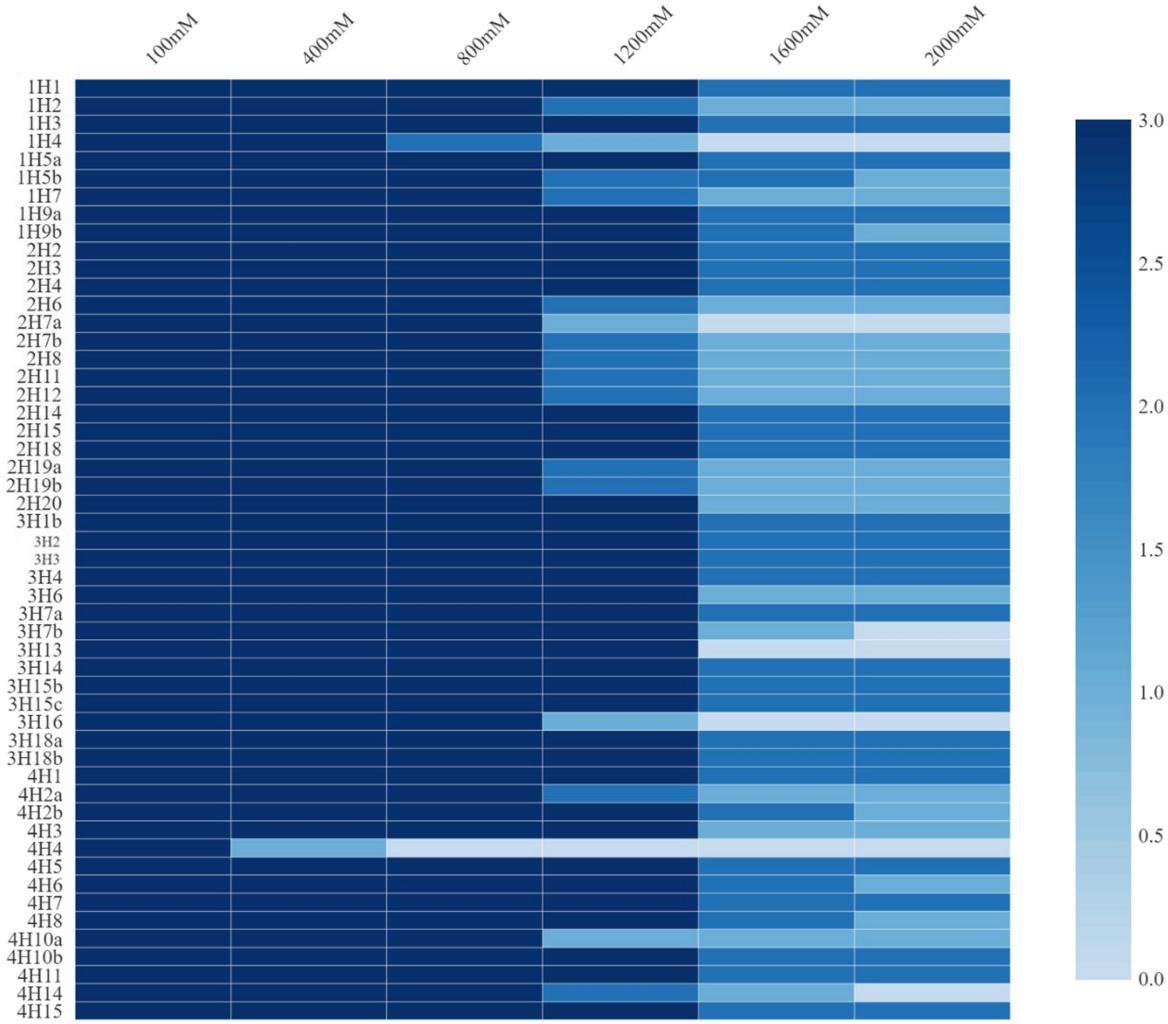
Salt tolerance levels of bacterial endophytes isolated from four different halophytes from Runn of Kutch.

These endophytes were subjected to growth under added salt concentrations of up to 2000 mM NaCl (Figure 1). The salt tolerance among the endophytes was very high and was almost similar in the isolates from all four plants. Growth up to 2000 mM NaCl in nutrient agar plates was recorded. In halophyte *Aeluropus* sp. (3H), the highest number of salt tolerant endophytes were found, followed by *Cressa cretica* (4H), and *Suaeda palmirosa* (1H).

**Figure 2:**
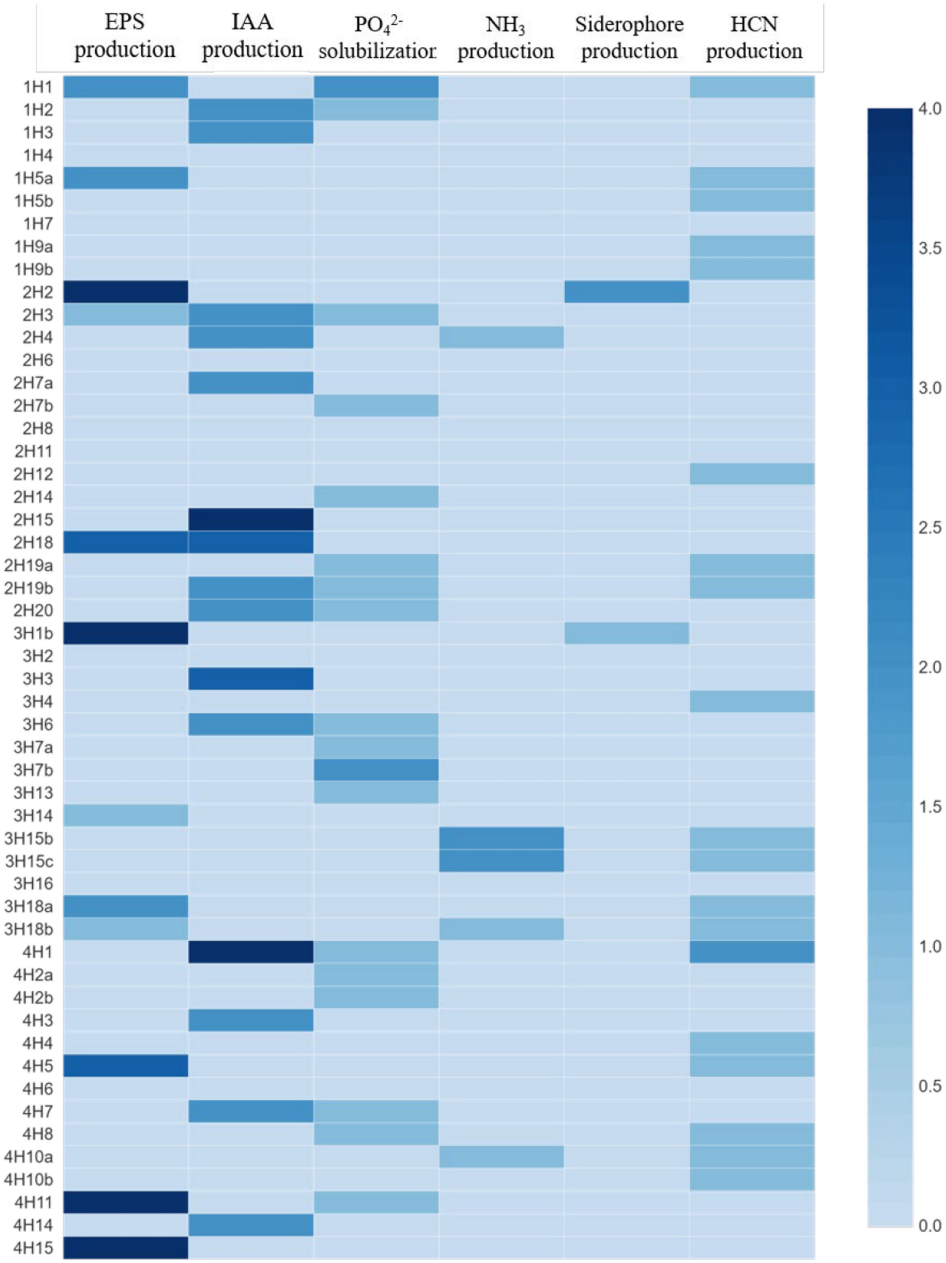
Plant growth promoting and stress alleviating features of bacterial endophytes isolated from four different halophytes from Runn of Kutch; Weightage in heat map: For PSB, and siderophore production: 0 = <10 mm, 1 = 10–12 mm, 2 = 12–14 mm, 3 = 14–16 mm, and 4 = >16 mm (size of clear zone). For NH_3_ and EPS production: 0 = no production, 1 = low, 2 = medium, 3 = high, and 4 = very high. For IAA production: 0 = no production, 1 = up to 5 μg/ml, 2 = 5–10 μg/ml, 3 = 10–15 μg/ml, and 4 = >15 μg/mL.

Among all the isolates, the highest IAA production was found in 2H15 (17.36 μg mL^−1^) and 4H1 (15.6 μg mL^−1^), followed by 3H3 (10.08 μg mL^−1^) (Figure 2). Looking at the functional diversity for IAA-producing endophytes (>5 μg mL^−1^) among four different halophytes, *Suaeda nudiflora* contained the highest IAA producers (66.67%), followed by *Aeluropus* sp. (42.85%), *Cressa cretica* (28.57%) and least was from *Suaeda palmirosa* (22.22%).

In the EPS production, the maximum production was in treatments 2H2, 3H1B, 4H11, and 4H15, followed by 4H5, 3H18a, 2H18, 1H5a, and 1H1 (Figure 2). Looking at the functional diversity for EPS-producing endophytes, the highest was in *Aeluropus* sp. (28.57%), and ~ 20% in the other three plant species.

In the phosphate solubilization potential, among all the isolates, 1H1 and 3H7b were the best phosphate solubilizers followed by 4H11, 4H8, and 4H7 (Figure 2). Looking at the functional diversity for phosphate solubilizing producing endophytes, the highest population was found in *Suaeda nudiflora* and *Cressa cretica* (~40%) followed by *Suaeda palmirosa* and *Aeluropus* sp. (~20%).

The maximum NH_4_ production was found in 3H15b and 3H15c treatments followed by 2H4, 3H18b, and 4H10a (Figure 2). Among the four halophytes, Only *Aeluropus* sp. was harboring good ammonia producers (21.43%), whereas, in *Suaeda nudiflora* and *Cressa cretica*, ~7% population was having Ammonia production ability. *Suaeda palmirosa* endophytes did not show any detectable ammonia production.

In the case of siderophore production, 2H2 and 3H1b were the best siderophore-producer inoculants (Figure 2). Among the halophytes, the siderophore producers were found in *Suaeda nudiflora* and *Aeluropus* sp. (~7%), whereas endophytes of *Suaeda palmirosa* and *Cressa cretica* we are not having any detectable siderophore production.

Opposite to the siderophore production, the HCN producer diversity was highest in *Suaeda palmirosa* (55.56%) and *Cressa cretica* (42.86%), followed by *Aeluropus* sp. (35.71%) and *Suaeda nudiflora* (20%) (Figure 2). Among the isolates, the significantly highest HCN-producing bacteria was 4H1 followed by 1H1, and 1H5a.

**Figure 3:**
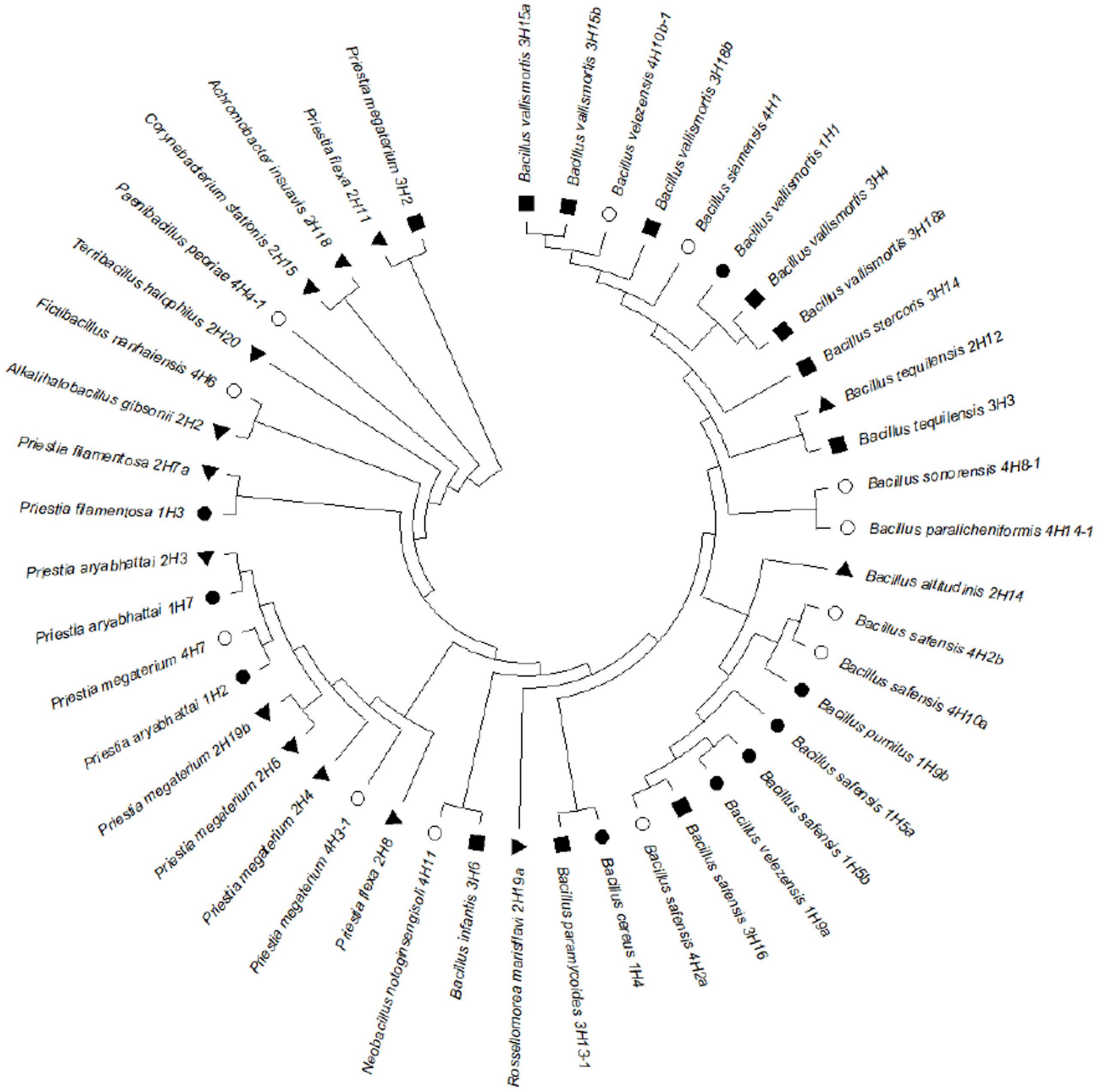
Phylogenetic tree showing relatedness of the bacterial endophytes isolated from four different halophytes from Runn of Kutch, signs represent the host halophytes: (●) *Suaeda palmirosa*, (▲) *Suaeda nudiflora*, (■) *Aeluropus* sp., and (○) *Cressa cretica*.

The 16S rRNA-based identity revealed that these endophytes belonged to genera *Bacillus, Priestia, Alkalihalobacillus, Corynebacterium, Achromobacter, Rossellomorea, Terribacillus, Paenibacillus, Fictibacillus*, and *Neobacillus* (Figure 3). The phylogenetic analysis showed that the distribution of the isolated endophytes in four halophytic plants has a pattern and most of the endophytes from one host were clustering together, possibly due to their close association through evolution. After removing duplicity, thirty-nine bacterial endophytes were submitted to National Agriculturally Important Microbial Culture Collection (NAIMCC) by the accession numbers NAIMCC-B-02945 to NAIMCC-B-02977, and NAIMCC-B-02990 to NAIMCC-B-02995.

### Plant growth parameters under stress

Based on multiple traits, four potential endophytes from these halophytic plants, *Alkalihalobacillus gibsonii* 2H2, *Achromobacter insuavis* 2H18, *Terribacillus halophilus* 2H20, and *Bacillus siamensis* 4H1 were tested in tomato, along with saline control. Growth induction was apparent from the plants. The parameters for salinity stress alleviation were found influencing by the endophyte inoculation. Total chlorophyll was highest in *Terribacillus halophilus* 2H20 (1.28 U mg^−1^ FW) inoculated plants followed by 2H18 (1.11 U mg^−1^ FW; Table 1), and the least was found in T1 control plants (0.80 U mg^−1^ FW). Plant height was significantly higher in T4 (2H20; 64.5 cm), T2 (2H2; 61.54 cm), and T3 (2H18; 59.63 cm) plants. The least was observed from saline control (52 cm). The highest dry biomass accumulation in the root was found in T4 inoculation (2.03 g) followed by T3 (1.73 g) and T5 (1.63 g). In the case of shoot dry weight, T4 inoculation (10.37 g) followed by T5 (8.74 g) and T2 (8.40 g) was having higher dry weight.

**Table 1:** Effect of endophytes isolated from four different halophytes from Runn of Kutch on growth parameters of tomato.

Treatment	Treatment details	Chlorophyll content	Plant height	Root dry weight	Shoot dry weight
T1	Control +150 mM NaCl	0.80e ± 0.04	52.00c ± 2.65	0.67d ± 0.13	6.63c ± 0.59
T2	*Alkalihalobacillus gibsonii* 2H2 + 150 mM NaCl	0.91d ± 0.02	61.54ab ± 2.33	1.39c ± 0.13	8.40b ± 0.26
T3	*Achromobacter insuavis* 2H18 + 150 mM NaCl	1.11b ± 0.03	59.63ab ± 2.66	1.73b ± 0.09	8.07b ± 0.38
T4	*Terribacillus halophilus* 2H20 + 150 mM NaCl	1.28a ± 0.01	64.50a ± 1.32	2.03a ± 0.09	10.37a ± 0.4
T5	*Bacillus siamensis* 4H1 + 150 mM NaCl	0.96c ± 0.02	57.26bc ± 4.39	1.63b ± 0.13	8.74b ± 0.38

The treatment data having same alphabets, are statistically non-significant at *p* ≤ 0.05 and means are separated by Duncan’s multiple range test. Errors values indicate standard deviation.

**Figure 4:**
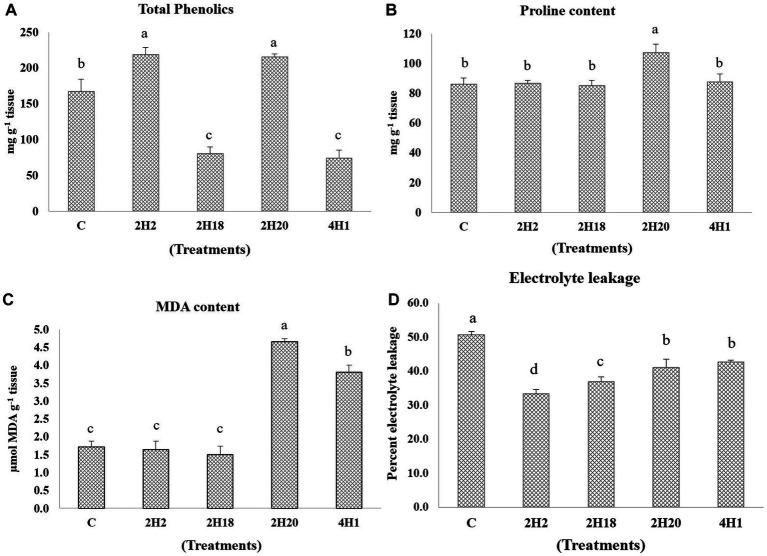
Effect of bacterial endophytes isolated from halophytes of Runn of Kutch on alleviation of ill effects of salinity in tomato plants. **(A)** Total phenolics, **(B)** Proline content, **(C)** MDA content, and **(D)** Electrolyte leakage. Treatments: C = Control +150 mM NaCl, 2H2 = *Alkalihalobacillus gibsonii* 2H2 + 150 mM NaCl, 2H18 = *Achromobacter insuavis* 2H18 + 150 mM NaCl, 2H20 = *Terribacillus halophilus* 2H20 + 150 mM NaCl, 4H1 = *Bacillus siamensis* 4H1 + 150 mM NaCl. The treatment data having same alphabets, are statistically non-significant at *p ≤ 0.05* and means are separated by Duncan’s multiple range test. Error bars indicate standard deviation.

Changes in the levels of antioxidants were analyzed. In total phenolics, 2H2 (218.89 mg g^−1^ tissue) and 2H20 (215.09 mg g^−1^ tissue) we are having the highest accumulation and the lowest were recorded in 4H1 (74.26 mg g^−1^ tissue) (Figure 4). The accumulation of proline was highest in the *T. halophilus* 2H20 inoculated plants. The significantly highest accumulation of proline was found in T4 (107.23 mg g^−1^ tissue) followed by T5 (87.53 mg g^−1^ tissue), T2 (86.53 mg g^−1^ tissue), and T1 (86.03 mg g^−1^ tissue), the lowest was recorded in T3 (85.03 mg g^−1^ tissue). The highest MDA content was observed from 2H20, followed by 4H1 (Figure 4). Control, 2H2, and 2H18 were having significantly low MDA content. In the case of electrolyte leakage, all the inoculated treatments were having low leakage. Reduction in electrolyte leakage was also observed from inoculation of *Suaeda nudiflora* and *Cressa cretica* endophytes, in T1 (50.65%) and T5 (42.65%), respectively. The lowest electrolyte leakage was observed in the leaves of *Alkalihalobacillus gibsonii* 2H2 inoculated plants.

**Figure 5:**
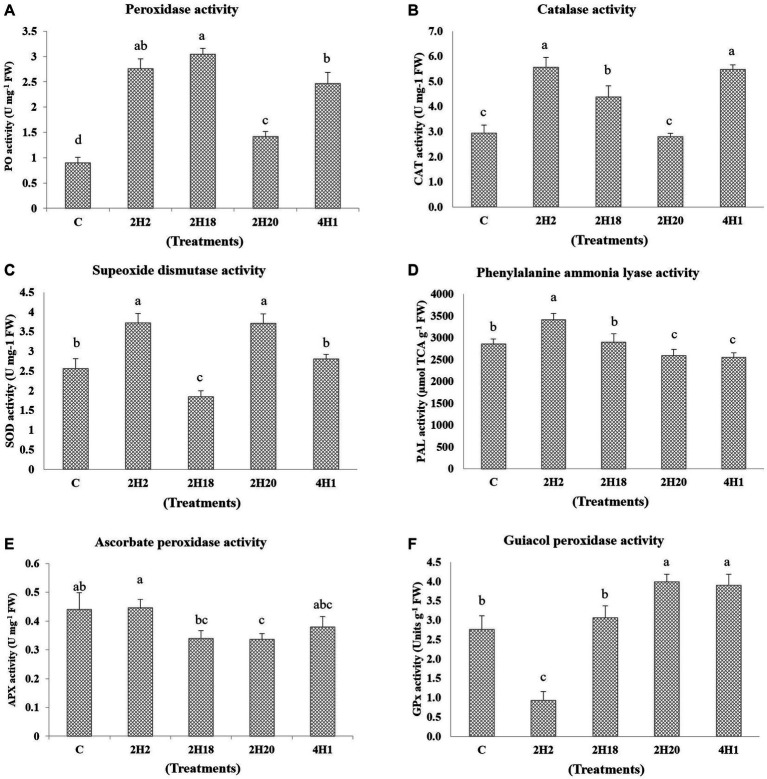
Effect of bacterial endophytes isolated from halophytes of Runn of Kutch on accumulation of enzymatic antioxidants in tomato under salinity. **(A)** Peroxidase activity, **(B)** Catalase activity, **(C)** Superoxide dismutase activity, **(D)** Phenylalanine ammonia lyase, **(E)** Ascorbate peroxidase activity, and **(F)** Guiacol peroxidase activity. Treatments: C = Control +150 mM NaCl, 2H2 = *Alkalihalobacillus gibsonii* 2H2 + 150 mM NaCl, 2H18 = *Achromobacter insuavis* 2H18 + 150 mM NaCl, 2H20 = *Terribacillus halophilus* 2H20 + 150 mM NaCl, 4H1 = *Bacillus siamensis* 4H1 + 150 mM NaCl. The treatment data having same alphabets, are statistically non-significant at *p* ≤ 0.05 and means are separated by Duncan’s multiple range test. Errors bars are indicating standard deviation.

The accumulation of enzymatic antioxidants was found to be enhanced by the halophytic endophyte inoculation (Figure 5). In the case of ascorbate peroxidase activity, T2 (0.45 U mg^−1^ FW) recorded the highest accumulation among all the treatments and the least was found in T4 (0.33 U mg^−1^ FW). However, the ascorbate peroxidase activity was highest in 2H2, 4H1, and control plants. Peroxidase accumulation was highest in 2H2 and 2H18 inoculated plants. The maximum PO accumulation was in T3 (3.04 U mg^−1^ FW) followed by T2 (2.76 U mg^−1^ FW) and T5 (2.47 U mg^−1^ FW). The least concentration was found in T1 (0.90 U mg^−1^ FW). The activity of guiacol peroxidase was highest in 2H20 and 4H1 inoculated plants. In GPX, we see maximal accumulation in T4 (3.99 U g^−1^ FW) followed by T5 (3.90 U g^−1^ FW) and T3 (3.06 U g^−1^ FW). The least was recorded in T2 (0.93 U g^−1^ FW). In the case of Catalase, T2 (5.57 U mg^−1^ FW) and T5 (5.48 U mg^−1^ FW) inoculated plants recorded higher accumulation followed by T3 (4.38 U mg^−1^ FW). The lowest recorded was from T1 (2.95 U mg^−1^ FW) and T4 (2.80 U mg^−1^ FW). In the case of superoxide dismutase, both 2H2 and 2H20 were having statistically significant accumulation. The highest accumulation of SOD was found in T2 (3.72 U mg^−1^ FW) and T4 (3.71 U mg^−1^ FW) which was significantly higher than T5 (2.80 U mg^−1^ FW) and T1 (2.56 U mg^−1^ FW). Significantly higher PAL concentration was recorded from T2 (3413.62 μmol TCA g^−1^ FW) followed by T1 (2861.62 μmol TCA g^−1^ FW) and T3 (2898.75 μmol TCA g^−1^ FW). The least was recorded from T4 (2587.09 μmol TCA g^−1^ FW) and T3 (2898.97 μmol TCA g^−1^ FW). This indicates the oxidative stress alleviation potential of the halophytic endophytes in tomato.

**Figure 6:**
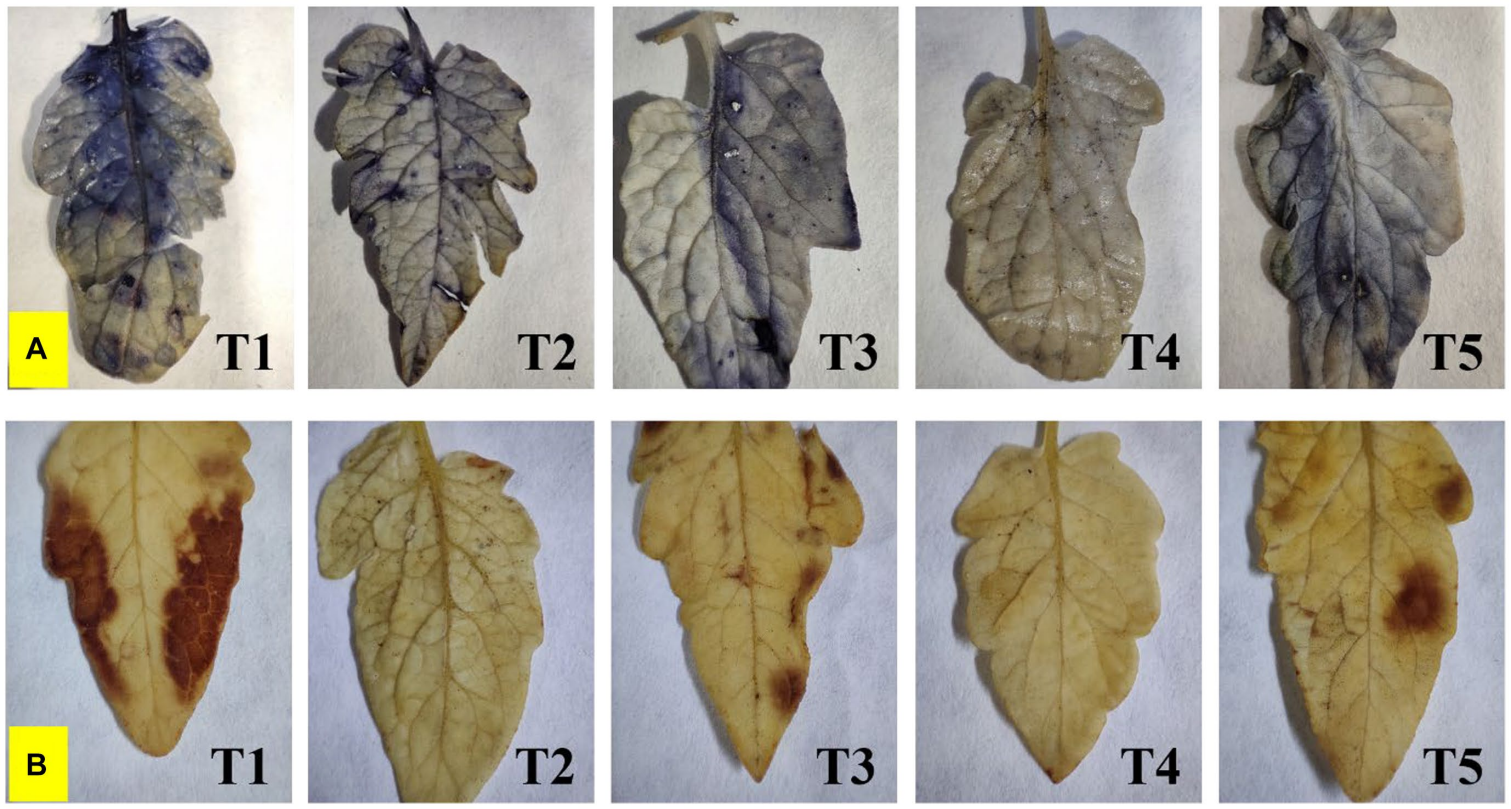
Effect of bacterial endophytes on **(A)** superoxide accumulation by NBT staining, and **(B)** salinity induced sub-cellular H_2_O_2_ accumulation by DAB staining under elevated salinity (150 mM NaCl); T1 = Control +150 mM NaCl, T2 = *Alkalihalobacillus gibsonii* 2H2 + 150 mM NaCl, T3 = *Achromobacter insuavis* 2H18 + 150 mM NaCl, T4 = *Terribacillus halophilus* 2H20 + 150 mM NaCl, T5 = *Bacillus siamensis* 4H1 + 150 mM NaCl.

Endophyte inoculation also reduced superoxide accumulation as visualized by NBT staining, and also has affected salinity-induced sub-cellular H_2_O_2_ accumulation as visualized by DAB staining (Figure 6). The superoxide accumulation was lowest in T4 plants, whereas the highest accumulation was seen in control plants. Sub-cellular H_2_O_2_ accumulation was lowest in T4 and T2 plants, whereas the highest was reported from control plants.

**Figure 7:**
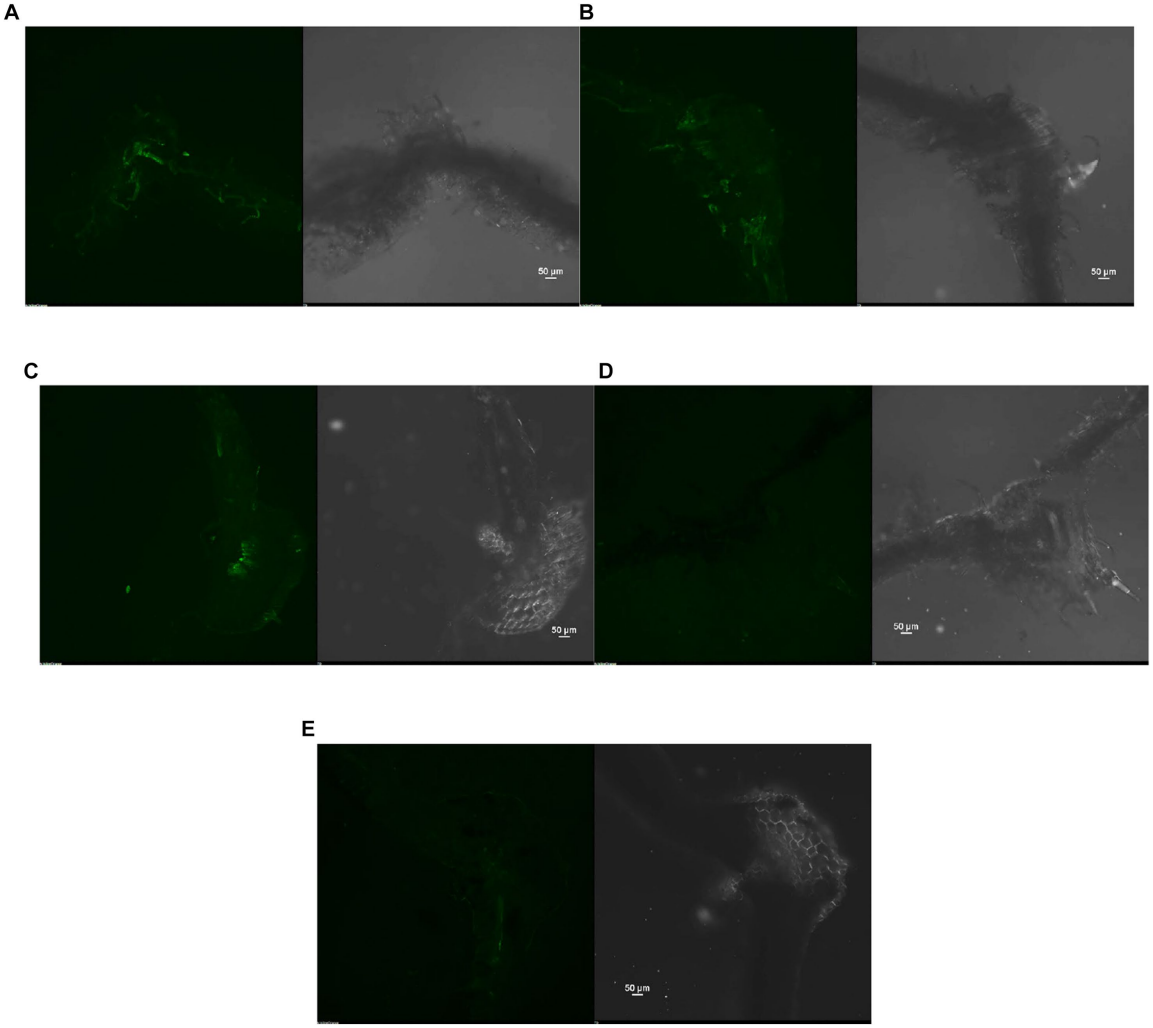
Confocal Scanning laser microscopic images showing difference in sodium accumulation in cross section of tomato leaves, **(A)** T1-Control +150 mM NaCl, **(B)** T2-*Alkalihalobacillus gibsonii* 2H2 + 150 mM NaCl, **(C)** T3-*Achromobacter insuavis* 2H18 + 150 mM NaCl, **(D)** T4-*Terribacillus halophilus* 2H20 + 150 mM NaCl, **(E)** T5-*Bacillus siamensis* 4H1 + 150 mM NaCl.

**Figure 8:**
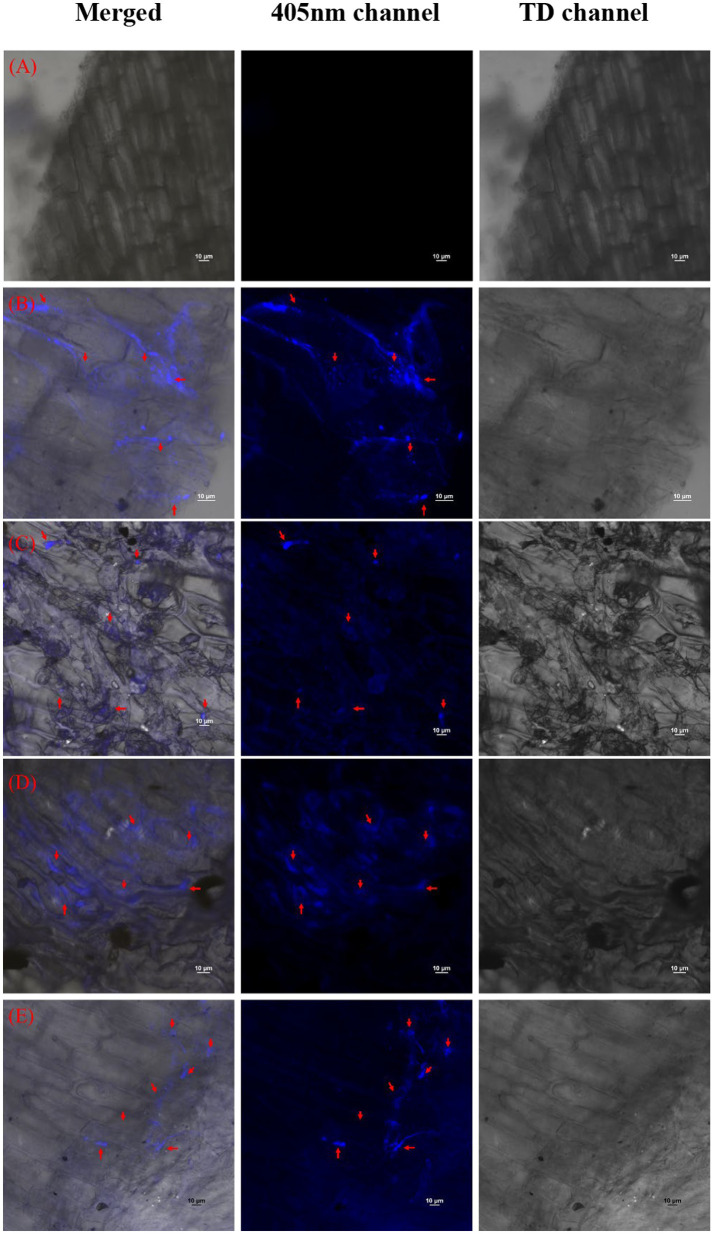
Confocal Scanning laser microscopic images showing colonization of halophytic endophytes in tomato roots in merged, 405 nm, and TD channels. Red arrows indicate clusters of inoculated bacterial cells with cell tracker blue **(A)** T1-Control +150 mM NaCl, **(B)** T2-*Alkalihalobacillus gibsonii* 2H2 + 150 mM NaCl, **(C)** T3-*Achromobacter insuavis* 2H18 + 150 mM NaCl, **(D)** T4-*Terribacillus halophilus* 2H20 + 150 mM NaCl, **(E)** T5-*Bacillus siamensis* 4H1 + 150 mM NaCl.

The reduction in the accumulation of Na^+^ ions in tomato leaves was visualized using SodiumGreen™ probes under CSLM and found to be lowest in *Bacillus siamensis* 4H1 inoculated plants (Figure 7). The xylem vessels of control plants were brightly illuminated indicating higher sodium transport and accumulation. In endophyte-inoculated treatments, the fluorescence was relatively less. The cross-genera colonization of halophyte endophytes was successful in tomato roots. The confocal scanning laser micrographs indicated the presence of cell tracker-treated inoculum of all four endophytes in tomato roots after 48 h of treatment (Figure 8). The highest colonization was observed in T2. In the control treatment, there were no signals from the cell tracker.

Summarily, in this study, we have explored the culturable endophytic diversity of four halophytic plants from Runn of Kutch for salt tolerance and plant growth promotion. Some of the endophytes could grow to 2000 mM of added NaCl; however, the pattern of tolerance was different in different hosts, where *Aeluropus* sp. Endophytes exhibited the highest tolerance. These endophytes were having varied growth promoting features that indicate toward possibility of coordinated approach for providing an overall plant stand to its host by complementing each other. The in-plant inoculation of tomato showed several successful cross-genera colonization. These potential colonizers were tested in tomato plants and were able to supplement plant stress mitigation mechanisms. These bacteria were able to increase oxidative stress mitigation and the accumulation of compatible solutes while reducing excess Na^+^ ions in plants and electrolyte leakage. However, the mode of action of these endophytes was different, indicating their complementary roles inside the plants and a higher stress tolerance.

## Discussion

In this work, we have identified moderately halophilic bacterial endophytes from halophytic plants grown in Runn of Kutch that could enhance plant growth in salt-stress situations. Plant growth and growth under various abiotic stress conditions were demonstrated to be significantly benefited by the inoculation of efficient halophilic bacterial endophytes (Sahu et al., 2021). Several endophytes have been reported to have the innate ability to deal with salinity stress and enhance stress resilience in plant growth when inoculated with plants in saline conditions (Egamberdieva et al., 2019). In our study, we also selected endophytes from halophytic plants with good tolerance to NaCl. One of the important aspects of this study was to screen the functional diversity of the selected endophytes so that they could be used as biostimulants to stimulate plant growth in saline environments under field conditions. These endophytes were able to produce excessive amounts of exopolysaccharides (EPS). Under salt stress conditions, the synthesis of EPS is crucial and forms a chelate with ionic metals (Na^+^, Cl^−^). It relieves salinity stress on plants by directly binding and immobilizing cations (Na^+^). The reduced Na^+^ uptake by the rice plant could be due to increased EPS synthesis by endophytic strains in the inoculated plant, thus identifying a key area for crop improvement. The high level of EPS production by endophytes enhanced biofilm formation and improved soil aggregation. For instance, Upadhyay et al. (2011) showed that wheat treated with bacterial culture under salt conditions significantly improved its growth properties.

IAA-producing endophytes were very common in these halophytes. However, the IAA producers were more common in *Suaeda nudiflora* which could be connected to the host growth-inducing mechanism of these endophytes. Studies revealed that increased salt stress had an impact on plants’ xylem and phloem concentrations of IAA (Junghans et al., 2006). Moreover, IAA is crucial for plant-microbe interactions, which may become unstable under various abiotic conditions (such as salt stress) (Spaepen and Vanderleyden, 2011). Thus, by producing more IAA under saline conditions and delivering it to the rhizosphere, salt-tolerant endophytes could promote the growth of salt-sensitive plants. The PGP characteristics of bacteria have been researched to identify those with a high potential for usage as biostimulants.

Very few endophytic bacterial isolates could produce ammonia in this study. Ammonia-producing bacteria are frequently reported to be able to provide ammonia as a nitrogen source for plant growth. Through the production of ammonia from the hydrolysis of urea into ammonia and carbon dioxide, bacterial endophytes can promote plant growth (Li et al., 2016). The reduced availability of ammonia producers could be due to their different nutrient requirements than the media used for isolation.

In this study, similar to ammonia production, the siderophore producers were also relatively less abundant in cultural diversity. This might be due to the presence of other mechanisms of siderophore reaction than what we have used in the modified CAS agar assay. Some of the isolates were good siderophore producers. These siderophore-producing isolates may help iron-deficient plants in enhancing growth and biomass accumulation (Khan et al., 2006). Additionally, bacterial strains produced hydrogen cyanide (HCN), a desirable component of plant growth promoting rhizobacteria (PGPR). Some rhizobacteria prevent the growth of plant diseases and boost the host’s disease-resistance system by producing HCN (Sahu et al., 2019).

Most of the isolated endophytes had a varying ability to solubilize phosphate. Inoculating *P*-solubilizing endophytic bacteria into the soil with limited phosphate availability results in improved plant growth performance (Wang et al., 2018). Previous studies also suggest an improvement in phosphorus uptake and overall plant growth has been reported through the use of endophytes as biostimulants (Khan and Doty, 2009). Phosphate solubilization by the endophytes under investigation may be important for their establishment in the rhizosphere. Previous reports have described the establishment of *Alkalihalobacillus gibsonii* in the rhizosphere of maize (Batool et al., 2019), *Achromobacter insuavis* in rice (Sukweenadhi et al., 2019), *Terribacillus halophilus* in *Vitis vinifera* (Salomon et al., 2016), and *Bacillus siamensis* in maize (Zhang et al., 2022).

In tomato, the increase of NaCl is closely associated with an increase in antioxidant enzyme activity (Zhang et al., 2018). To limit the harm caused by salt stress, plants have evolved an enzyme defense mechanism. The results of the current study clearly show a significant increase in proline content and antioxidant enzyme activity in plants treated with endophytic strains. The findings imply that more antioxidant activity is necessary at higher NaCl doses to protect plants from oxidative damage brought on by salinity (Sahu et al., 2021). One of the most prevalent alterations under salt stress is proline accumulation in plant cells. Results showed that the 2H20 has higher proline accumulation, which corresponds to improved stress tolerance in 2H20-inoculated plants. Proline is a key osmoprotectant that shields bacterial cells from the osmotic stress caused by salinity stress (Zhang et al., 2018). It is therefore an essential solute that accumulates in microorganisms and plants to restore normal activities and is significantly more abundant in stressed plants than in healthy ones. This might help to explain why certain physiological systems in plants are activated in salinity stress situations (Sahu et al., 2022). The effect of the bacterial strain’s inoculation is indicated in the current study by the greater concentration of proline content in inoculated plants compared to non-inoculated plants. This study revealed that plants may be able to reduce oxidative damage brought on by salt through the action of bacteria (Sahu et al., 2021; Gupta et al., 2023a). Additionally, Lipid peroxidation (based on MDA content) had a trend like proline content.

Results showed that the endophyte inoculation reduced the electrolyte leakage from leaves. Ion-specific salt damage primarily affects plasma membranes (Katuwal et al., 2020). In order to identify salt-tolerant plants, electrolyte leakage from plasma membranes is described as one of the most crucial selection criteria (Sahu et al., 2021). Increases in salt concentration led to a rise in electrolyte leakage for all inoculated treatments. In this case, high EL values might be caused by a naturally high potassium level instead of being a sign of damage to the plasma membrane caused by stress, as Rivero et al. (2022) explain. Similar results have been reported from our previous studies (Sahu et al., 2021; Gupta et al., 2023a). According to Gupta et al. (2023a), the loss of chlorophyll in salt-stressed plants is a common sign of oxidative stress. This is because the enzyme chlorophyllase stops the plant from making chlorophyll and speeds up its breakdown. A photoprotection mechanism was suggested where the declining chlorophyll contents were either due to sluggish synthesis or rapid chlorophyll breakdown. In the *in planta* study, four endophytic bacterial strains were examined for their effects. Endophytes, or bacteria that live inside eukaryotic hosts, face challenging conditions because of a lack of available nutrients, space, and control over cellular growth from their hosts. The endophytic cell may experience simultaneous bursts of oxidation and the production of ROS. Increased antioxidant enzyme activity reduces oxidative stress. Endophyte survival within the host is thereby made possible. These antioxidants also help plants in ameliorating the ill effects of oxidative stress arising from salinity stress (Sahu et al., 2021). Various physiological and morphological factors that promote plant growth were examined after the incubation of plants with each of these isolates to identify the most efficient endophyte.

Our results suggest that CAT, APX, and GPX activities, when coordinated with SOD activities, play a major protective role in the O_2_ and H_2_O_2_ scavenging process and that the active participation of these enzymes is linked, at least in part, to tomato plants’ tolerance to salt-induced oxidative stress. Inoculation of endophytes enhanced the activity of antioxidant enzymes, except for APx. Plants use a variety of enzymatic and non-enzymatic antioxidants to maintain reactive oxygen species concentrations within a specific functional range in order to prevent oxidative harm (Sahu et al., 2021). Antioxidant enzymes like catalase, superoxide dismutase, and peroxidase have been shown to reduce the levels of superoxide and hydrogen peroxide in plants that are under stress from salt (Sahu et al., 2022). It was discovered that the high-yielding allele had greater activity of the antioxidant enzymes CAT APX and GPX. Additionally, CAT and APX are the most efficient antioxidant enzymes for averting cell damage, according to (Kumar et al., 2019). These two enzymes demonstrated a critical role in controlling intracellular amounts of H_2_O_2_. In plants, over-expression of the SOD and CAT genes has been shown to enhance defense against reactive stress (Gupta et al., 2023a).

The reduced biomass may be caused by the higher Na^+^ accumulation. In this experiment, the highest biomass accumulation in root and shoot was observed in 2H20 inoculated plants which was corresponding to the confocal images of sodium accumulation in leaves. The biomass of plants declined with rising salinity, according to previous research (Sahu et al., 2021). The ion toxicity, osmotic stress, and oxidative stress altogether decline plant productivity. By neutralizing lipid free radicals or stopping the conversion of hydroperoxides into free radicals, phenolic compounds show antioxidant activity (Sahu et al., 2022). The ability of plants to generate antioxidants regulates the extent of cellular oxidative harm in plants under abiotic stress. Therefore, the rise in plant antioxidant levels to detoxify the reactive oxygen species generated in these circumstances appears to be favoring salt tolerance. Increased production of enzymatic and non-enzymatic antioxidants could be responsible for reduced oxidative stress in plants. Staining for H_2_O_2_ and superoxide accumulation also supported these findings. Other reports also suggest that the microbes which are able to directly or indirectly reduce either of these effects could ameliorate salinity stress in plants (Gupta et al., 2023a,b).

These endophytes were successfully colonized in tomato roots. This could be the first step in providing salinity amelioration to the plants. The colonization of microbes in the roots can be studied using fluorescent-based dyes. One such popular dye is BacLight™. The BacLight™ has been initially used for purposes like monitoring the permeabilization of starter cells and the lysis of *Lactococcus lactis* in cheese using a confocal microscope (Bunthof et al., 2001). There were a few reports studying the endospore viability of pathogens after gamma-irradiation using BacLight™ (Laflamme et al., 2004). The most common use of BacLight™ was in flow cytometry for studying live-dead cells after certain treatments (Berney et al., 2007). monitoring live and dead bacteria in bioaerosols to assess the human health threat due to allergens and toxins produced by the bacteria present in the air (Nasrabadi et al., 2018). There were reports of colonization of endophytes in plants of genera different than their host plants (Sahu et al., 2021) using BacLight™ dye. The Blue CMAC dye used in the present investigation provides a rather specific movement study of the bacterial cells in the plant system.

The CellTracker™ Blue CMAC dye has been earlier used for tracking a single genus in systems like water, as reported by Fuller et al. (2000) for *Comamonas* sp. strain DA001 in water. In some of the studies, this dye was used to mark the mitochondria and vacuole while studying the degradation of mitochondria by autophagy (Mao et al., 2013). However, the use of CellTracker™ Blue CMAC dye for studying root colonization in a different genus was not reported earlier. This result is more specific and accurate as compared to the BacLight™ dye for studying the movement of a single microbial strain. These results could be beneficial in further widening the host range of potent microbial cultures from any crop to any crop.

The endophytes of halophytic plants had multiple plant growth-promoting traits under elevated salinity. Endophyte *Terribacillus halophilus* 2H20 performed best among four potential endophytes that could be further utilized for making formulations of stress-alleviating bacteria.

Climate change and increasing population pressure on available farmlands have compelled the development of strategies for climate-resilient agricultural practices. Microbial biostimulants are one such brilliant agent that could support plant mechanisms to cope with environmental stresses and enhance crop productivity (Castiglione et al., 2021). Plant performance is determined by the plant genome and the associated microbiome (Ali et al., 2022). Endo-microbiome engineering for increasing the population of stress-tolerant microbes could be a robust strategy for developing climate-resilient agriculture. Our present study showed that some of the stress-tolerant endophytes from halophytic plants could colonize tomato plants and provide stress tolerance to the new host plant. Since developing stress-tolerant genotypes has several limitations, stress-tolerant endophytes that have wider host adaptation can be explored with greater utility in transferring stress tolerance.

## Conclusion

In order to reach sustainable food production, climate-resilient cropping strategies should be explored. Microbial inputs are proven to have salinity stress tolerance. Thus, in the current study, we have explored endophytes from Runn of Kutch. The findings of the present study conclude that there is the possibility of using endophytes from halophytic plants to inoculate commercial crops and could provide salinity stress tolerance by multiple mechanisms. The endophyte *Terribacillus halophilus* 2H20 was the most promising isolate. The halophytic endophytes showed differential activation of antioxidant molecules, possibly indicating operation of different mechanisms of salinity stress alleviation, e.g., Na^+^ exclusion, compartmentalization, Na^+^/K^+^ balance, production of compatible solutes, alleviation of oxidative stress, etc. The endophytes having different mechanisms could be further explored to collectively inoculate on the same plants for their successful colonization and functioning in the plants as a designer microbiome to achieve better plant health and growth.

## Data availability statement

The datasets presented in this study can be found in online repositories. The names of the repository/repositories and accession number(s) can be found in the article/Supplementary material.

## Author contributions

PSa: conceptualization, software, data curation, writing—original draft preparation, and visualization. PSa, SS, ZS, KO, KJ, JT, and NM: methodology. PSa and SS: validation. PSh and AS: formal analysis. PSa, SS, and ZS: investigation. PSa, PSh, and AS: resources and writing—review and editing. All authors have read and agreed to the published version of the manuscript.

## Funding

This research was funded by Indian Council of Agricultural Research (ICAR), under NBAIM project “Consortium of endophytic and rhizospheric bacteria for alleviation of salinity stress in tomato” grant number IXX14218. The APC was funded by ICAR.

## Conflict of interest

The authors declare that the research was conducted in the absence of any commercial or financial relationships that could be construed as a potential conflict of interest.

## Publisher’s note

All claims expressed in this article are solely those of the authors and do not necessarily represent those of their affiliated organizations, or those of the publisher, the editors and the reviewers. Any product that may be evaluated in this article, or claim that may be made by its manufacturer, is not guaranteed or endorsed by the publisher.

## Footnotes

^1^https://www.ezbiocloud.net/

^2^https://www.ncbi.nlm.nih.gov/
