# Supplementary material for: Colonization potential of endophytes from halophytic plants growing in the “Runn of Kutch” salt marshes and their contribution to mitigating salt stress in tomato cultivation
Source: Front Microbiol. 2023 Aug 29;14:1226149. doi: 10.3389/fmicb.2023.1226149 (PMC10495581; doi:10.3389/fmicb.2023.1226149)
Supplement: Supplementary file 1 [file Table_1.DOCX]

The sequencing data has been deposited in NCBI and the live cultures are deposited at National Agriculturally Important Culture Collection and International Depository Authority (NAIMCC), India. The accession numbers are given in the Table S1.

**Table S1: Accessions of halophytic endophytes submitted to NCBI and NAIMCC**

| **Sr. No** | **Endophytes** | **NCBI accession** | **NAIMCC accession** | **Sr. No** | **Endophytes** | **NCBI accession** | **NAIMCC accession** |
| --- | --- | --- | --- | --- | --- | --- | --- |
|  | *Bacillus vallismortis* 1H1 | OK178867 | NAIMCC-B-02960 |  | *Priestia megaterium* 3H2 | OK178884 | NAIMCC-B-02974 |
|  | *Priestia aryabhattai* 1H2 | OK178868 | NAIMCC-B-02968 |  | *Bacillus tequilensis* 3H3 | OK178885 | NAIMCC-B-02959 |
|  | *Priestia filamentosa* 1H3 | OK178869 | NAIMCC-B-02970 |  | *Bacillus vallismortis* 3H4 | OK178886 | NAIMCC-B-02961 |
|  | *Bacillus cereus* 1H4 | OK178870 | NAIMCC-B-02948 |  | *Bacillus infantis* 3H6 | OK178887 | NAIMCC-B-02949 |
|  | *Bacillus safensis* 1H5a | OK178871 | NAIMCC-B-02952 |  | *Bacillus paramycoides* 3H13-1 | OL589557 | NAIMCC-B-02951 |
|  | *Priestia aryabhattai* 1H7 | OL639226 | NAIMCC-B-03093 |  | *Bacillus stercoris* 3H14 | OK178889 | NAIMCC-B-02957 |
|  | *Bacillus pumilus* 1H9b | OL639228 | NAIMCC-B-03090 |  | *Bacillus safensis* 3H16 | OK178890 | NAIMCC-B-02953 |
|  | *Alkalihalobacillus gibsonii* 2H2 | OK178872 | NAIMCC-B-02946 |  | *Bacillus siamensis* 4H1 | OK178891 | NAIMCC-B-02955 |
|  | *Priestia aryabhattai* 2H3 | OK178873 | NAIMCC-B-02969 |  | *Bacillus safensis* 4H2a | OK178892 | NAIMCC-B-02954 |
|  | *Priestia megaterium* 2H4 | OK178874 | NAIMCC-B-02973 |  | *Bacillus safensis* 4H2b | OL639234 | NAIMCC-B-03091 |
|  | *Priestia megaterium* 2H6 | OL639229 | NAIMCC-B-03095 |  | *Priestia megaterium* 4H3-1 | OL589551 | NAIMCC-B-02975 |
|  | *Priestia filamentosa* 2H7a | OK178875 | NAIMCC-B-02971 |  | *Paenibacillus peoriae* 4H4-1 | OL589552 | NAIMCC-B-02967 |
|  | *Priestia flexa* 2H8 | OK178877 | NAIMCC-B-02972 |  | *Bacillus vallismortis* 4H5-1 | OL589553 | NAIMCC-B-02962 |
|  | *Priestia flexa* 2H11 | OL639230 | NAIMCC-B-03094 |  | *Fictibacillus nanhaiensis* 4H6 | OK178896 | NAIMCC-B-02965 |
|  | *Bacillus tequilensis* 2H12 | OK178878 | NAIMCC-B-02958 |  | *Bacillus sonorensis* 4H8-1 | OL589555 | NAIMCC-B-02956 |
|  | *Bacillus altitudinis* 2H14 | OK178879 | NAIMCC-B-02947 |  | *Bacillus safensis* 4H10a | OL589556 | NAIMCC-B-03092 |
|  | *Corynebacterium stationis* 2H15 | OK178880 | NAIMCC-B-02964 |  | *Bacillus velezensis* 4H10b-1 | OL589549 | NAIMCC-B-02963 |
|  | *Achromobacter insuavis* 2H18 | OK178881 | NAIMCC-B-02945 |  | *Neobacillus notoginsengisoli* 4H11 | OK178899 | NAIMCC-B-02966 |
|  | *Rossellomorea marisflavi* 2H19a | OK178882 | NAIMCC-B-02976 |  | *Bacillus paralicheniformis* 4H14-1 | OL589550 | NAIMCC-B-02950 |
|  | *Terribacillus halophilus* 2H20 | OK178883 | NAIMCC-B-02977 |  |  |  |  |
